# 2605. Etiology of Community Acquired Lower Respiratory Tract Infection (LRTI) in Children

**DOI:** 10.1093/ofid/ofad500.2219

**Published:** 2023-11-27

**Authors:** Byungsun Yoo, Ilha Yune, Eu Suk Kim, Sungyoon Lim, Sooyoung Yoo, Seyoung Jung, Miyoung Kim, Junesung Kim, Daehwan Kim, Hyunju Lee

**Affiliations:** Seoul National University Bundang Hospital, Seongnam, Kyonggi-do, Republic of Korea; Seoul National University Bundang Hospital, Seongnam, Kyonggi-do, Republic of Korea; Seoul National University Bundang Hospital, Seongnam, Kyonggi-do, Republic of Korea; Seoul National University Bundang Hospital, Seongnam, Kyonggi-do, Republic of Korea; Seoul National University Bundang Hospital, Seongnam, Kyonggi-do, Republic of Korea; Seoul National University Bundang Hospital, Seongnam, Kyonggi-do, Republic of Korea; Seoul National University Bundang Hospital, Seongnam, Kyonggi-do, Republic of Korea; Planit Healthcare, Seongnam, Kyonggi-do, Republic of Korea; Planit Healthcare, Seongnam, Kyonggi-do, Republic of Korea; Seoul National University Bundang Hospital, Seongnam, Kyonggi-do, Republic of Korea

## Abstract

**Background:**

Community acquired lower respiratory tract infection (LRTI) is a leading cause for hospitalization in children and an important cause for antibiotics. Despite the large burden of LRTI, in many cases it is difficult to determine the pathogen. In this study, we aimed to describe the etiology of LRTI in children and analyze factors associated with bacterial or viral infection.

**Methods:**

Electronic medical records of children < 19 years of age diagnosed with LRTI (including pneumonia, bronchiolitis or bronchitis) at Seoul National University Bundang Hospital from January 2005 to June 2019 were retrospectively reviewed.

**Results:**

A total of 5,954 cases of LRTI were identified. Among them 74.3% were pneumonia and 25.7% were bronchiolitis or bronchitis. Mean age was 22 (IQR 38) months. 52.6% were < 2 years of age and 29.7% were 2-4 years of age. Among all LRTI, 48% had a proven pathogen; 55.1% were viral, 46.3% were *Mycoplasma pneumoniae* and 1.3% were bacterial. Among viruses, respiratory syncytial virus (41.9%) was most common followed by influenza (7.8%), parainfluenza (7.1%) and adenovirus (4.1%). Among bacteria pneumonia, *Streptococcus pneumonaie* was most common which accounted for 58.1%. Among physical findings, wheezing, stridor, chest retraction and coarse breathing sounds were seen more commonly in viral infections (P< 0.05, respectively), whereas decreased breathing sound was commonly found in both *M. pneumoniae* and bacterial infections (P< 0.05, respectively). For treatment, 26.5% were treated with supportive care, 72.0% received antibiotics, 1.0% received antiviral agents and 5.6% were prescribed with steroids. Among patients 16.4% needed oxygen supply, 1.7% were admitted to the intensive care unit and 1.4% required respiratory support. There was one case of mortality (0.02%).

**Conclusion:**

In children with LRTI, among cases where the pathogen was detected, more than half were due to viral infections, followed by *M. pneumoniae*, and a few cases of bacteria. In this study, we found a relatively large portion of children may have been treated with unnecessary antibiotics. LRTIs in children are a significant clinical burden and calls for a need for antibiotic stewardship.

**Disclosures:**

**All Authors**: No reported disclosures

